# Supplementary material for: Morphology and Molecular Phylogeny of Four Anaerobic Ciliates (Protista, Ciliophora, Armophorea), with Report of a New Species and a Unique Arrangement Pattern of Dikinetids in Family Metopidae
Source: Microorganisms. 2025 Jan 23;13(2):240. doi: 10.3390/microorganisms13020240 (PMC11857559; doi:10.3390/microorganisms13020240)
Supplement: Supplementary file 1 [file microorganisms-13-00240-s001.zip › Table S1.pdf]

Table S1. The GenBank accession numbers that are not shown in the phylogenetic tree (Figure 9).

Species name	Accession number	Species name	Accession number
Brachonella contorta	KX776449	*Brachonella contorta*	KX776475
Brachonella contorta	KX776450	*Brachonella contorta*	KX776475
Brachonella contorta	KX776451	*Metopus es*	KX776453
Brachonella contorta	KX776452	*Metopus es*	KX776459
Brachonella contorta	KX776454	*Metopus es*	KX776462
Brachonella contorta	KX776455	*Metopus es*	KX776463
Brachonella contorta	KX776456	*Metopus es*	KX776464
Brachonella contorta	KX776458	*Metopus es*	KX776465
Brachonella contorta	KX776460	*Metopus es*	KX776469
Brachonella contorta	KX776466	*Metopus es*	KX776470
Brachonella contorta	KX776467	*Metopus es*	KX776472
Brachonella contorta	KX776468	*Metopus es*	KX776474
Brachonella contorta	KX776471	*Metopus es*	KX776476
Brachonella contorta	KX776473	*Metopus es*	LN869960
